# Do digital health interventions hold promise for stroke prevention and care in Black and Latinx populations in the United States? A scoping review

**DOI:** 10.1186/s12889-023-17255-6

**Published:** 2023-12-21

**Authors:** Bianca D. Rivera, Claire Nurse, Vivek Shah, Chastidy Roldan, Adiebonye E. Jumbo, Mohammad Faysel, Steven R. Levine, David Kaufman, Aimee Afable

**Affiliations:** 1 School of Public Health, Downstate Health Sciences University, 450 Clarkson Avenue, Brooklyn, NY 11203 USA; 2 College of Medicine, Downstate Health Sciences University, 450 Clarkson Avenue, Brooklyn, NY 11203 USA; 3 School of Health Professions, Health Informatics Program, Downstate Health Sciences University, 450 Clarkson Avenue, Brooklyn, NY 11203 USA; 4 Department of Neurology/Stroke Center, Downstate Health Sciences University, 450 Clarkson Avenue, Brooklyn, NY 11203 USA

**Keywords:** Stroke, Digital health, Health equity, Telehealth, Telestroke, mHealth, Mobile health

## Abstract

**Background:**

Black and Latinx populations are disproportionately affected by stroke and are likely to experience gaps in health care. Within fragmented care systems, remote digital solutions hold promise in reversing this pattern. However, there is a digital divide that follows historical disparities in health. Without deliberate attempts to address this digital divide, rapid advances in digital health will only perpetuate systemic biases. This study aimed to characterize the range of digital health interventions for stroke care, summarize their efficacy, and examine the inclusion of Black and Latinx populations in the evidence base.

**Methods:**

We searched PubMed, the Web of Science, and EMBASE for publications between 2015 and 2021. Inclusion criteria include peer-reviewed systematic reviews or meta-analyses of experimental studies focusing on the impact of digital health interventions on stroke risk factors and outcomes in adults. Detailed information was extracted on intervention modality and functionality, clinical/behavioral outcome, study location, sample demographics, and intervention results.

**Results:**

Thirty-eight systematic reviews met inclusion criteria and yielded 519 individual studies. We identified six functional categories and eight digital health modalities. Case management (63%) and health monitoring (50%) were the most common intervention functionalities. Mobile apps and web-based interventions were the two most commonly studied modalities. Evidence of efficacy was strongest for web-based, text-messaging, and phone-based approaches. Although mobile applications have been widely studied, the evidence on efficacy is mixed. Blood pressure and medication adherence were the most commonly studied outcomes. However, evidence on the efficacy of the various intervention modalities on these outcomes was variable. Among all individual studies, only 38.0% were conducted in the United States (*n* = 197). Of these U.S. studies, 54.8% adequately reported racial or ethnic group distribution. On average, samples were 27.0% Black, 17.1% Latinx, and 63.4% White.

**Conclusion:**

While evidence of the efficacy of selected digital health interventions, particularly those designed to improve blood pressure management and medication adherence, show promise, evidence of how these interventions can be generalized to historically underrepresented groups is insufficient. Including these underrepresented populations in both digital health experimental and feasibility studies is critical to advancing digital health science and achieving health equity.

**Supplementary Information:**

The online version contains supplementary material available at 10.1186/s12889-023-17255-6.

## Introduction

Stroke is a leading cause of serious long-term disability and death in the United States (U.S.) [1, 2]. Despite declines in stroke mortality in the general U.S. population, Black and Latinx individuals lag and suffer more from adverse stroke-related outcomes compared with Whites, including higher rates of recurrent stroke. After declining for decades and stalling in recent years, stroke prevalence and mortality trends increased again in 2020 [3, 4]. These rates are expected to continue to increase [5], given both an aging population and the impact of the COVID-19 pandemic. COVID-19 survivors are at a 52% increased risk of stroke 1-year post-infection [6], with younger people at risk and unaware of stroke symptoms [7]. Black Americans are currently at the highest risk of mortality, with Latinx men projected to have the highest increase in stroke mortality rates by 2030 [5].While the reasons for this disparity are complex, studies show that Black and Latinx stroke survivors have persistently worse risk factor profiles relative to White stroke survivors, including higher rates of uncontrolled hypertension, diabetes, and hyperlipidemia [8]. Relative to White individuals, Black and Latinx people are also more likely to lack health insurance, have no routine interactions with healthcare providers, or have forgone or delayed medical care due to cost. These are vital markers that influence or impact the success of continuity of care [9].

Fragmented healthcare systems present major challenges to the continuity of care required by clinic-based stroke prevention and rehabilitation solutions. There is a promise that digital solutions may reduce these barriers. Recent evidence shows an impressive expansion of mobile health or home-based support solutions for stroke care across different digital modalities [8, 10–12]. Yet our ability as a society to care for the most vulnerable has not kept up with this speed of innovation for several reasons.

The digital divide, which follows historic disparities in health, is a major barrier to advancing the field of digital health. In the U.S. for example, nearly 21 million people do not have broadband internet access [13], a necessary connection for many digital health interventions. Inequities in broadband access mirror those in health: Black and Latinx individuals, those living in poverty, and rural areas all disproportionately lack access [14–17]. Recent novel theoretical frameworks of the digital divide and health equity also acknowledge a myriad of additional reasons for its existence: differences in digital literacy, self-efficacy, attitudes toward technology, implicit tech bias and algorithmic bias, to name a few [14, 18, 19]. Without deliberate attempts to reduce the digital divide, rapid advances in digital health will only perpetuate systemic biases and widen the gap [20, 21]. For example, uptake of telehealth solutions during the pandemic varied along racial and ethnic lines, with Black patients having higher odds of going to the emergency room, urgent care, and in-person office visits compared to White patients who leveraged telehealth [22, 23].

At the same time, the underrepresentation of historically excluded populations in scientific discovery is well-documented. Lack of diversity is seen in a wide range of clinical trials [24–28], genome-wide association studies (GWAS) [29], and in data mining of electronic health records [20, 21, 30]. This underrepresentation of populations of color in research is due to historical patterns in the way these populations have been engaged, if at all, in the research process. First because these populations are typically not consulted when defining the research question, which is a requirement in community participatory research, there is no explicit value placed on their expertise nor a reciprocal transfer of knowledge [31]. Second, there is a history of mistrust in scientific studies and in the healthcare system more broadly that results in underrepresentation of these groups in scientific discovery [32–34].

It is unclear what aspects of stroke care digital interventions would produce the most profound benefits for patients, caregivers, and health care providers [35]. As a fundamental step toward achieving health equity and advancing the science of digital health, a scoping review was initiated with the goal of using the findings to design a digital health intervention prototype that is acceptable and feasible for populations that have been historically excluded. As such, Transcreation [36], an implementation science framework that places equal value on community practice and scientific evidence, served as a theoretical basis for organizing our scoping review. The aims of our scoping review follow the intervention design steps of Transcreation, which include the selection of promising inputs for the intervention prototype by reviewing evidence-based interventions and examining their commonalities. First, we aim to identify and characterize the range of digital health interventions for stroke care in the peer-reviewed evidence base. We define these interventions broadly as information and communications technologies (ICT) in medicine and other health professions to manage illness, health risks, and promote wellness [37]. ICTs are tools and resources to transmit, store, create, share, or exchange information [38]. Digital health has a broad scope, and common interventions include wearable devices, mobile health, telehealth, health information technology, and telemedicine [37]. Second, we summarize efficacy findings for the identified digital health intervention modalities. Third, we are required to design an intervention prototype to fit the community setting and population. To do so, we must examine the extent to which existing evidence includes historically excluded populations. As such, our scoping review also aims to characterize the global diversity of individual studies included across the literature and among studies with U.S. participants, characterize their demographic and geographic diversity.

## Methods

### Data sources and search strategy

We searched PubMed, the Web of Science, and EMBASE for publications between January 1, 2015 and June 8, 2021, pertaining to our target population, digital interventions, and systematic reviews. Key search terms and the iterations that followed center around “stroke,” “hypertension,” “diabetes,” “smoking,” “hyperlipidemia”, in conjunction with various interventions including but not limited to “mobile applications,” “text messages,” “virtual reality,” and systematic reviews of experimental or intervention studies. The complete search strategy is provided in Additional file Table 1.

Our choice to begin the search as of 2015 is supported in two ways. First, this work serves as a practice-based follow-up to an Agency for Healthcare Research and Quality (AHRQ) 2016 report on telehealth and patient outcomes [39]. While the AHRQ report search ended on January 30, 2016, the last publication year in the synthesis was 2015 (for only one study). Second, the report followed the previous precedent of excluding telephone-only interventions from the definition of ‘telehealth.’ Upwards of 50 reviews pertaining to our target population were excluded in their review, with this intervention modality potentially serving as the exclusionary factor. As outlined in the criteria below, we are not excluding telephone-only as this modality may be effective for our target population, especially considering the digital divide. Given the speed of innovation, we believe 2015 as a search year provides a balance between previous review limitations.

### Inclusion criteria

The following inclusion and exclusion criteria were established a-priori. Reviews were included if they were (1) a peer-reviewed systematic review or meta-analysis of (2) experimental, quasi-experimental, or intervention studies (3) assessing the impact of any digital health intervention, including multi-modality interventions on (4) risk factors for stroke and outcomes related to physical dimensions of stroke prevention and rehabilitation in (5) adults age 18 years and older. We did not restrict on intervention setting or comparator group. Specific risk factors for stroke include hypertension, type 2 diabetes, high cholesterol, and smoking. Reviews targeting these populations were included, as were reviews targeting individuals with chronic diseases or cardiovascular conditions, given the significant overlap in modifiable risk factors and the prevalence of co-morbidity between them. Both diagnosed and at-risk healthy populations were included as well.

Reviews were excluded initially for any of the following criteria: (1) reviews that did not use a documented systematic search (e.g., narrative review), (2) solely included pediatric or adolescent populations, (3) assessed mental health or non-clinical outcomes (e.g., quality of life, patient satisfaction, cost-effectiveness), (4) did not present stratified findings to synthesize for this review (e.g., general “mHealth,” “eHealth,” “tele-rehabilitation”). Lastly, studies were excluded if (5) they were not in the English language.

We implemented a second selection phase after reviewing conflicts at the full-text review stage of screening. This was necessary given the importance of understanding the scope of potential benefits digital interventions may have for stroke-related care. For reviews with diverse populations (multiple chronic diseases, children and adults) and study designs, we established the following three additional criteria for a total of eight exclusion criteria: (6) for broad health reviews, majority of individual studies did not focus on stroke or risk factors for stroke, where majority is defined as greater than 50% of all synthesized research within a review, (7) for reviews with broad study design inclusion, majority of individual studies were not experimental or intervention-based, or (8) for reviews including individual studies without age restriction, sample mean age < 18.

### Data extraction

The nine study team members collaborated on all screening, review, and extraction phases. Reviews from the database searches were imported into *Covidence* [40], a web-based collaboration software platform that streamlines the production of systematic and other literature reviews. Both title and abstract screening and full-text review were completed in *Covidence*, which tracked inclusion/exclusion consensus and flagged conflicts across both review stages. Consensus between two independent reviewers was required before proceeding to the next stage. The team met virtually to discuss conflicts related to inclusion. The extraction template was created by an iterative and collaborative process using included studies to ensure accurate data capture. Study team members divided the extraction process, and each study was extracted by one team member. Extraction progress was discussed during team meetings, where additional decisions were made if necessary. Data of interest to extract included: digital modality, clinical area, target population, functionality/strategy, publication search range, number of original publications included in the synthesis by type of design, primary and secondary outcomes, and results.

The first author manually reviewed all individual studies synthesized in each systematic review for data collection pertaining to geographic diversity and racial/ethnic and gender representation for studies conducted in the U.S. We classified the reporting of racial/ethnic distribution across studies as adequate reporting, inadequate reporting, and not reporting. Adequate reporting includes studies where 100% of the sample's racial/ethnic distribution is described. Inadequate reporting refers to studies where (1) the sample is only described by the greatest proportion of racial/ethnic group membership (i.e., 70% White, or 70% Black), without describing the remaining racial/ethnic group distribution, or (2) authors aggregate the remaining distribution into a large “other group” (i.e., 70% White, 30% Non-White). We classified studies as not reporting racial/ethnic distribution if this information was missing from the sample description in the methods, results, or manuscript tables.

### Efficacy evaluation

We developed a three-tiered system to summarize the strength of efficacy findings for the identified digital health intervention modalities. A strong case for potential benefits overall between the modality and a given health outcome occurred when (1) a meta-analysis found a significant effect in the experimental group or (2) the majority (> 50%) of individual studies within a review found a significant benefit when a meta-analysis was not conducted. Findings were deemed inconclusive when ≤ 50% of the studies within a review found a significant effect. This indicates that studies may have found positive significant effects in favor of modality, negative effects, or no significant effects. Lastly, if no studies found a significant effect or if a meta-analysis was performed and no effect was found, we determined that there was no overall benefit by the modality for a particular outcome. If the meta-analysis aggregated only by function (e.g., monitoring) or outcome (e.g., blood pressure) and not modality, then we did not report meta-analytic findings.

### Quality assessment

Critical appraisal of all included research was assessed using the ten criteria established for summarizing systematic reviews by Aromataris et al. [41]. Using the systematic review or meta-analysis as the analytical unit of review, we evaluated the review research question, inclusion criteria, search strategy, sources (databases) for search, their critical appraisal process, analysis/synthesis, recommendations and assessment of limitations. A full list of the criteria can be found in Additional file Table 2. The first appraisal was conducted by the study team member extracting the data. The first author conducted the second appraisal.

## Results

**Fig. 1:**
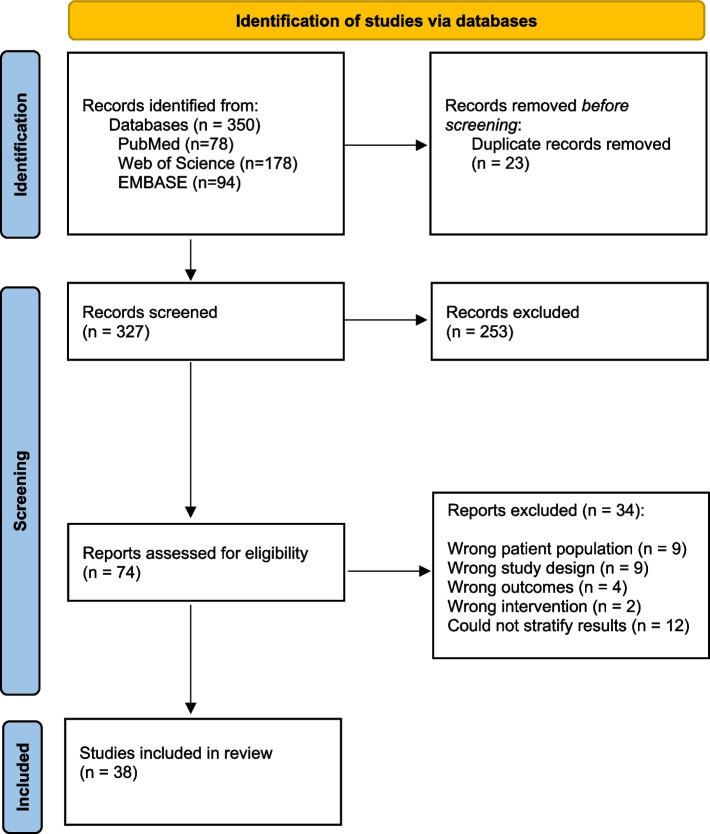
Study flow diagram using the PRISMA model

The search strategy resulted in 350 systematic reviews or meta-analyses, which were screened across titles and abstracts. Of these, 74 were selected for a full-text review, and 38 met the full criteria for this scoping review (Fig. 1).

### Quality of reviews

The quality of the 38 selected reviews was assessed across ten criteria (Additional file Table 2) [41]. One review did not meet three criteria (appropriate inclusion criteria, critical appraisal reviewed by two authors, and recommendations for practice) [42]. Six reviews did not meet two criteria [43–48]. Twelve reviews did not meet one criterion. Most often, publication bias was not assessed (*n* = 13, 34.2%), followed by critical appraisal not being conducted by two independent reviewers (*n* = 7, 18.4%). The overall risk of bias was low across all reviews and criteria (30/38, 78.9%).

### Characteristics of included reviews

Descriptive characteristics of the 38 review articles are summarized in Table 1, with Additional file Table 3 providing details for each review article. Most review criteria included people with stroke (*n* = 15, 39.5%) or type 2 diabetes (*n* = 13, 34.2%). Other samples included people with hypertension, high cholesterol, gestational diabetes, broad cardiovascular risk, and people who smoke, as well as a general at-risk population. These health domains were not mutually exclusive.

**Table 1:** Scope of reviews included in synthesis (*n* = 38)

	**n (%)**
**Health Domains**	
Stroke	15 (39.5)
Type 2 Diabetes	13 (34.2)
Hypertension	5 (13.2)
High Cholesterol	1 (2.6)
Smoking	2 (5.3)
Gestational Diabetes	1 (2.6)
Cardiovascular Diseases or CVD risk	4 (10.5)
At-risk general population	3 (7.9)
**Function Categories**	
Consultation	2 (5.3)
Education	15 (39.5)
Monitoring	19 (50.0)
Case Management	24 (63.2)
Mentoring	5 (13.2)
Rehabilitation	11 (28.9)
**Digital Modality**	
Computer-delivered	2 (5.3)
Web-based	13 (34.2)
Messaging	9 (23.6)
Telestroke	2 (5.3)
Mobile applications	13 (34.2)
Health devices	9 (23.6)
Virtual reality	8 (21.1)
Phone-based	6 (15.8)

Reviews can explore more than one health domain, functionality, or modality. Categories are not mutually exclusive and will add to greater than 100%

*CVD* Cardiovascular disease

Cardiovascular diseases or CVD risk includes, for example, “cardiovascular diseases,” ischemic heart disease, myocardial infarction, acute coronary syndrome, heart failure, peripheral arterial disease, and “mixed level of cardiovascular risk.” These studies also included other stroke risk factors

‘At-risk general population’ includes studies whose sample is comprised of adults 18 + years of age [49, 50], 19 + years of age [51], and children [50]

Digital interventions were used for six tele-strategies, identified and defined by Lee et al. [52], with the addition of a newly proposed functional strategy: tele-rehabilitation for post-stroke care. Strategies are not mutually exclusive, as interventions may have multiple components designed for many functions. The most common strategies included case management (*n* = 24, 63.2%) and monitoring (including self-monitoring) (*n* = 19, 50%). Often these were used in combination together.

We discovered a total of eight modalities across all reviews. Many interventions consisted of multiple modalities; therefore, modalities were also not mutually exclusive. The list of modalities is as follows: computer-delivered, web-based, messaging, telestroke, mobile applications, health devices, virtual reality, and phone-based.

Web-based interventions require an internet connection delivered via an internet-enabled device and include the receipt of e-mail or access to web pages [47, 49, 51, 53–62]. While all mobile applications require an internet connection, the distinction for a web-based intervention is that the user is expected to access a web page or resource online. However, some interventions were designed with equity in mind and users could choose how to participate (i.e., web-based if they did not own a smartphone to access a mobile application) [54]. These interventions were used for education, monitoring, case management, and mentoring. Mobile applications are computer programs or software applications designed to run on a mobile device such as a phone, tablet, or watch [43, 47, 50, 53–56, 63–68]. Our review identified a range of functionalities associated with mobile applications including education, monitoring, case management, and mentoring.

Messaging-based interventions involved automated and interactive communication channels between the user and a healthcare professional, with some tailored to individual patients [43, 47, 48, 53, 54, 56, 57, 65, 68]. These interventions required the user to have a device to receive messages, which differs from web-based e-mail communication, which can be received on various devices but requires internet access. Messages could be received directly on a device (SMS) or through a mobile application. Automated messages may include reminders for medication adherence or physical activity. As such, they were used for education, case management, and mentoring. Phone-based interventions did not utilize messaging but audio-only phone calls for case management only [43, 51, 54, 55, 65, 68]. They often were used in addition to other modalities.

Health devices are equipped with sensory technology for health care monitoring as its primary function [43, 45–47, 54, 56, 57, 69–71]. They provide an opportunity to transfer physiological data, such as remote blood pressure monitors and glucometers. Devices may also report data related to medication adherence (e.g., digital medication trays) or assist with case management and rehabilitation (e.g., MusicGlove Hand Therapy) [72].

Modalities solely used for post-stroke care included computer-delivered, telestroke, and virtual reality. Telestroke is used for consultation by experienced healthcare providers who remotely care for people who have a stroke in another location [42, 73, 74]. Computer-delivered interventions require the use of a computer and may also often require access to a program or software for the intervention [53, 75]. The user cannot access the programming on a mobile device. Virtual reality (VR) is an advanced form of human–computer interface that allows the user to interact with and become immersed in a computer-generated environment [44, 76–82]. Varying levels of immersion (low-, semi-, full-) depend on the specific technology used, ranging from PC monitors (low), motion trackers (semi), or head-mounted displays (full). VR can be offered as either (1) “off the shelf” commercial video gaming consoles (e.g., Nintendo Wii) or (2) custom-built virtual environments, where the former is often used for recreational purposes. VR in medical settings is used for rehabilitation, including balance, mobility, cognitive, and motor functions [79].

The most common modalities across included reviews were web-based and mobile applications (*n* = 13, 34.2%, each) and messaging and health devices (*n* = 9, 23.6%, each).

### Synthesis of findings

Table 2 presents a synthesis of outcomes related to stroke or risk factors for stroke across the eight modalities. For each review, we determined the proportion of studies with results suggesting that the modality effectively improved an outcome. However, for meta-analyses by modality, we accepted the overall conclusion found from the reported analysis.

**Table 2:** Efficacy of digital health intervention modalities by outcome across all reviews

	Web-based	Messaging	Phone-Based (Calls/Audio-only)	Mobile Applications	Health Devices	Computer-delivered	Telestroke	Virtual Reality
**Clinical Outcome**								
HbA1c	[54, 58–60]	[47, 48, 54]		[47, 53, 56, 63–65] [66]	[46] [45]			
Fasting Blood Sugar	[60]	[47]		[65]				
Blood Pressure	[47, 58] [59] [55]	[47]	[55]	[67] [53, 66] [55]	[71]			
Anthropometry	[49, 51, 58, 60]		[51]	[50, 63, 67] [64] [53]				
Cholesterol	[58] [53] [59]			[53]				
Identifying Atrial Fibrillation					[69]			
**Post-Stroke Outcomes**								
Balance (e.g., Functional Reach, Berg Balance Scale)								[44, 77–80] [81]
Mobility/Activity (e.g., Timed Up & Go, Carrying, Walking)					[70]	[53]		[44, 78–81] [82]
Function (Joint, Muscle)					[70]	[53]	[42]	[76] [82]
Mortality							[42, 73]	
Symptomatic Intracranial Hemorrhage							[42, 73]	
Onset to Door/Onset to Treatment							[73]	
Length of Hospital Stay							[73] [42]	
Memory						[53] [75]		
Speech						[53]		
**Behavioral Outcomes**								
Medication Adherence	[55] [53]	[43, 48, 53, 68] [57]	[55, 68]	[63, 67] [68]				
Diet	[53, 60]			[53]				
Physical Activity	[53, 54, 58]	[54]		[54] [53] [50]				
Smoking Cessation	[61, 62]			[53]				

A solid circle indicates (1) a meta-analysis finds a significant effect in the experimental group or (2) the majority of individual studies find a significant benefit (> 50%) when a meta-analysis is not conducted. This indicates a strong case for potential benefits between the modality and the health outcome. A semi-solid circle indicates < 50% of the studies within a review find a significant effect. These findings would be inconclusive. A hollow circle indicates no studies found a significant effect or a meta-analysis was performed, and no effect was found. This would suggest no benefit by the modality upon the outcome

If the meta-analysis aggregated by function (e.g., monitoring) or outcome (e.g., blood pressure) and not modality, then we did not report meta-analytic findings in this table

Measures of anthropometry include BMI [49–51, 53, 63], body weight [49, 50, 58, 60, 64], and waist circumference [49, 64]

### Primary and secondary stroke prevention

#### Messaging, phone-based, and web-based interventions

While nine reviews explored interventions inclusive of messaging, only seven allowed us to synthesize results. Both Hyun et al. (2021) and Fu et al. (2017) had primary mobile applications combined with messaging and other secondary modalities [56, 65]. There was strong evidence that simple messaging interventions could significantly decrease HbA1c [47, 48, 54], fasting blood sugar [47], and blood pressure [47]. There was also strong evidence that messaging could increase physical activity [54] and medication adherence [43, 48, 53, 68]. However, one review also suggested there are inconclusive findings regarding the efficacy of messaging on medication adherence [57].

Like messaging, phone-based interventions significantly increased medication adherence [55, 68]. Additional evidence suggests that phone-based case management could significantly decrease blood pressure [55] and BMI [51].

Whereas thirteen reviews explored web-based interventions, only eleven allowed us to synthesize results. The excluded reviews reported the combination efficacy of modalities using web-based components [56, 57]. For clinical outcomes, there is strong evidence that web-based interventions can decrease HbA1c [54, 58–60], fasting blood sugar [60], and various measures of anthropometry, including body weight [49, 58, 60] and waist circumference [49, 64]. However, decreases in blood pressure and cholesterol levels may vary. While there is strong evidence in two reviews favoring web-based interventions decreasing blood pressure [47, 58], two reviews suggest inconclusive or no evidence [55, 59]. Similarly, one review suggests strong evidence for decreasing cholesterol [58], and two others suggest inconclusive or no evidence [53, 59]. Web-based interventions also showed a significant impact on behavioral outcomes such as physical activity [53, 54, 58], diet [53, 60], and medication adherence [55]. However, one review found no such evidence for medication adherence [53]. Lastly, web-based interventions were shown to be successful in increasing smoking cessation [61, 62].

#### Mobile applications & health devices

Mobile applications were used to evaluate both clinical and behavioral outcomes. There is strong evidence that mobile applications can decrease HbA1c [47, 53, 56, 63–65], fasting blood sugar [65], blood pressure [67], measures of anthropometry [50, 63, 67], and cholesterol [53]. However, other reviews found inconclusive evidence for changes in HbA1c [66], blood pressure [53, 66], and anthropometry [64]. Other reviews found no evidence that mobile applications can impact blood pressure [55] or anthropometry [53].

Mobile applications also increased medication adherence [63, 67], physical activity [54], and adherence to a healthy diet [53]. However, one review found inconclusive evidence that mobile applications could impact physical activity [53]. No evidence was found in three reviews—suggesting that mobile applications did not impact medication adherence [68], physical activity [50], or smoking cessation [53].

Health devices were used to evaluate various clinical outcomes, including HbA1c and blood pressure, and to identify atrial fibrillation. One review suggests strong evidence that health devices can significantly decrease HbA1c [46], and another suggests that blood pressure can also decrease [71]. However, one review found no evidence that health devices can impact Hba1c significantly [45].

### Tertiary stroke prevention & care

Health devices were also used for post-stroke rehabilitation. However, there is inconclusive evidence that health devices can increase mobility or function [70].

Computer-delivered interventions were used for post-stroke mobility, function, memory, and speech improvement. Though only one review synthesized the impact of mobility, function, and speech, there was strong evidence to suggest improvements in these areas [53]. Two reviews assessed memory—one indicating strong evidence [53] and the other inconclusive evidence [75].

Telestroke was used to evaluate post-stroke function (e.g., joints, limbs, muscles), mortality (in-hospital and 90-day), symptomatic intracranial hemorrhage (sICH), onset to door/onset to treatment, and length of hospital stay. There was strong evidence that telestroke could impact onset to door/onset to treatment and decrease the length of hospital stay [73]. However, one review found no evidence that telestroke could decrease the length of hospital stay [42]. This same review did not find evidence that telestroke could improve post-stroke function [42]. Both reviews did not find conclusive evidence that telestroke can improve post-discharge mortality or sICH.

Virtual reality was used to evaluate balance, mobility, and function post-stroke. While multiple reviews found strong evidence that balance could significantly approve post-stroke through virtual reality [44, 77–80], one review found no evidence of this benefit [81]. Multiple reviews also found strong evidence that mobility could be improved [44, 78–81], though one review found inconclusive evidence [82]. Few reviews evaluated function post-stroke; one found strong evidence [76], and another found inconclusive evidence [82].

### Diversity of studies selected into reviews

**Fig. 2:**
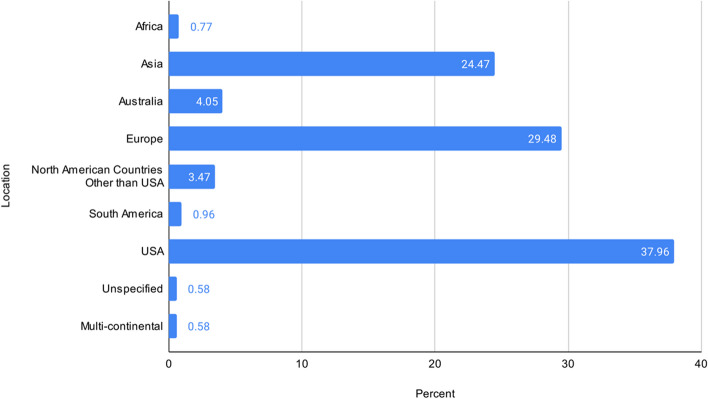
The proportion of study setting (*n* = 519) by continent. Note. This adds to more than 100%, since multiple studies were conducted internationally. Some studies occurred on multiple continents (e.g., web-based international samples). The U.S. is shown on its own to contrast the distribution of overall studies conducted in the U.S. vs. other areas

The 38 reviews yielded 519 unique studies published between 1986 and 2020. Most were only cited once across reviews (*n* = 405, 78.0%), ninety were cited twice (17.3%), and twenty-four were cited three or more times (4.6%). These studies represent 43 unique countries (41.4% North America, 1.0% South America, 29.5% Europe, 1.0% Africa, 24.5% Asia, and 4% Australia), of which 38.0% of all individual studies are from the U.S. (*n* = 197) (Fig. 2). Few studies were multi-continental (0.6%) or had an unspecified country/continent (0.6%). Since some studies were conducted across multiple continents, these values do not add to 100%.

We were particularly interested in the diversity across studies conducted in the U.S. Of the 197 total studies with U.S. participants, six included participants from other countries, and results were not stratified by setting. An additional three studies captured by the reviews did not have participants (i.e., the study was about theoretical usability, but no users were surveyed). A final total of 188 studies were used for the diversity analysis (Table 3). Most studies reporting location were conducted in the Southern (*n* = 46, 24.5%) and Western (*n* = 44, 23.4%) U.S. Some studies (*n* = 26, 13.8%) did not disclose location, and 16 studies (8.5%) were multi-state/national, but did not indicate the distribution of response by location. Multiple studies were conducted in the following research institutions/areas: Kaiser Permanente in California and Colorado, Duke University, Mayo Clinic (Rochester, MN), Boston, MA, and Pittsburgh, PA. Few studies characterized urbanicity; therefore, we cannot report on this factor.

**Table 3:** Diversity of individual studies conducted in the United States from included reviews (*n* = 188)

	**n (%)**
**Age**	
*Mean Age of Participants (years)*	
Under 18	5 (2.7)
18–24	6 (3.2)
25–34	4 (2.1)
35–44	26 (13.8)
45–54	40 (21.3)
55–64	59 (31.4)
65 +	35 (18.6)
% studies age not reported or mean not given	13 (6.9)
**Gender**	
Mean proportion female (%)	53.9
*Proportion female*	
0–20%	14 (7.5)
21–40%	23 (12.2)
41–60%	80 (42.5)
61–80%	50 (26.6)
81–100%	17 (9.1)
% studies gender not reported	4 (2.1)
**Race**	
% studies race not reported	44 (23.4)
% studies race reporting inadequate	41 (21.8)
% studies race reporting adequate	103 (54.8)
*Mean proportion White (n* = *92)*	*63.4*
*Mean proportion Black (n* = *95)*	*27.0*
*Mean proportion Latinx (n* = *52)*	*17.1*
*Mean proportion Asian (n* = *23)*	17.4
**Setting**	
*Region*	
Northeast	38 (20.2)
Midwest	30 (16.0)
South	46 (24.5)
West	44 (23.4)
*National, unspecified state*	16 (8.5)
*Unspecified*	26 (13.8)
**Digital Modalities**	
Computer-delivered	3 (1.6)
Web-based	109 (58.0)
Messaging	11 (5.9)
Telestroke	17 (9.0)
Mobile applications	14 (7.4)
Assistive device	7 (3.7)
Health devices	41 (21.8)
Virtual reality	4 (2.1)
Phone-based	39 (20.7)
Miscellaneous	4 (2.1)
Combination	45 (23.9)

Total US studies across reviews=197. However, six are multi-country and did not stratify age by country. Three additional studies captured by reviews do not have samples (i.e., about usability, but no users)

One study age was not reported, and 12 studies only report the distribution of participant age by cohorts, with age cohort bands differing between studies

**Fig. 3:**
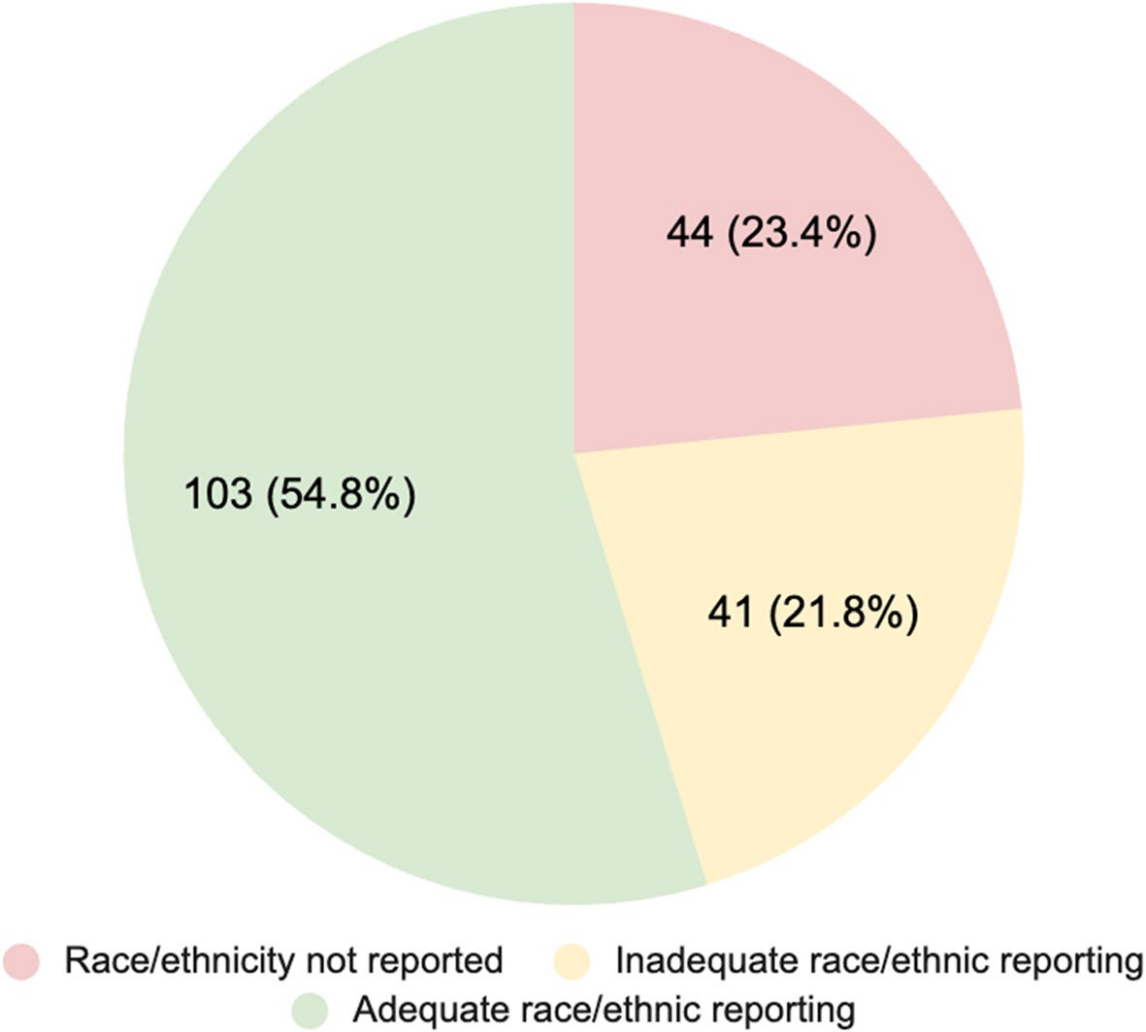
Adequacy of Reporting Race/Ethnic Group Distribution in U.S. Studies (*n* = 188)

We were also interested in demographic differences, such as age, gender, and race/ethnicity. Only 54.8% adequately reported racial/ethnic distribution of the sample, indicating all groups included, without aggregating small counts or defining the “Other” category. The remaining studies did not report race/ethnicity (*n* = 44, 23.4%) or inadequately did so (*n* = 41, 21.8%). For example, only indicating the group with the highest distribution (often White), aggregating non-White together, or aggregating low counts into an “Other” category arbitrarily. When race was reported, on average, 63.4% of the sample was White, 27.0% was Black, 17.1% Latinx, and 17.4% Asian (Fig. 3).

Most studies had a mean age of 55–64 years old (*n* = 59, 31.4%), followed by 45–54 years old (*n* = 40, 21.3%), though 13 studies did not report a mean (i.e., reported proportion of the sample within an age range), or age was not described. For studies reporting sex, on average 53.9% of participants were described as female.

Lastly, given the known efficacy of digital interventions across many outcomes, we were interested in capturing the frequency of modalities assessed in the U.S. Largely, these interventions were web-based (*n* = 109, 58.0%), used in combination with other modalities (*n* = 45, 23.9%), included health devices (*n* = 41, 21.8%), or were phone-based (*n* = 39, 20.7%).

## Discussion

This review had four aims. The first was to identify and characterize the range of digital health interventions for stroke care in the peer-reviewed evidence base. We found eight modalities that fit all of Lee et al.’s tele-strategies [52] and proposed adding tele-rehabilition as a strategy. The most common modalities across included reviews were web-based and mobile applications. The most common strategies included case management and monitoring (including self-monitoring). This confirms that digital interventions for stroke care and its risk factors are often leveraged for continuity of care for patients who are already at risk of stroke.

We then sought to summarize efficacy findings for the modalities. Interventions were commonly used for primary and secondary prevention or tertiary prevention. Web-based, phone-based, and messaging interventions show promise as low-resource digital solutions. At the same time, mobile applications and health devices also show promise, though as medium-resource digital solutions that require devices on hand (e.g., smartphones, glucometers). Nevertheless, five modalities have the potential to impact people at risk for stroke or risk factors for stroke. Health devices were also used for post-stroke care, as were computer-delivered interventions, telestroke, and VR. It should be noted that telestroke is not a patient-driven intervention, as it is used remotely in the acute-care setting by the patient’s care team or for follow-up care for patients unable to travel (i.e., in rehabilitation centers or nursing homes). These varying resource levels for modalities may impact acceptability and adoption for patients with varying levels of digital health literacy.

Finally, concerning the clinical and behavioral outcomes studied, adequate evidence supports the promise of digital health interventions designed to improve blood pressure and medication adherence. These were the most commonly studied outcomes, and about half of all reviews were conclusive with regard to efficacy. Notably, the evidence on the efficacy of remote device monitoring on blood pressure is conclusive based on the one review in our analysis. Similarly, evidence for diet and fasting blood sugar is conclusive, although these outcomes were less commonly studied.

While we are confident in the strength of these findings, some may need to be interpreted cautiously. For reviews that included multiple modalities or outcomes, we stratified results, such that there may be indicators of strong evidence when there may be 1 of 1 study that finds positive results (e.g., McLean et al. (2016), phone-based, blood pressure [55]). It is also important to note that significant findings across all reviews synthesized here may not indicate clinical significance. We are also confident that reviews with inconclusive or no evidence may not detract from the significance of positive findings. Reviews often cited small sample sizes as a limitation. These inherent limitations suggest that results may be subject to change if interventions are sufficiently powered.

For more complex interventions like VR, the many components of implementation (low-, semi-, full-immersion) may also impact variable findings. The review by Cheok et al. focused only on one type of low-immersion VR, the Nintendo Wii [81]. No evidence was found between the Nintendo Wii and balance improvement post-stroke. This review likely indicates that the components of the specific intervention may not benefit patients, while other forms of VR may be successful (semi- or full-immersion). Similarly, while there appears to be variability in the strength of research for web-based interventions and mobile applications, both modalities can be delivered in various ways. For example, differences in the delivery of the interventions may be dependent on the theoretical constructs driving the intervention and its functions, the user interface, dual-modal potential (e.g., optimizing the intervention for web and smartphone use), or the intervention ‘dose’ (how often are users accessing the website or the mobile application), how often should they be accessing?). Sufficient heterogeneity across these interventions may confound their true impact. Nevertheless, their overall success indicates that these interventions are at a minimum, feasible for users. However, future studies and systematic reviews in this field should consider theory and intervention components (elements of how the functionalities are delivered) and exhibit caution when aggregating results solely on modality.

We then explored the diversity of the studies synthesized in the 38 included reviews. With approximately 62% of studies conducted outside of the U.S., it raises the question if some of the interventions can be translated with similar outcomes to the U.S. population given its vastly different social and political landscape. Much of the innovation in this area (e.g., VR) does not seem to be studied in the U.S. Studies conducted in the U.S. commonly used web-based solutions (58%) or a combination of modalities. It raises concerns that 24% of the population lacks access to reliable broadband, while many low-resource solutions are conducted via the Web. However, the increased use of multiple modalities may make up for our fragmented care system, providing multiple digital touchpoints for patients and their care teams.

Lastly, among studies with U.S. participants, we characterized their demographic and geographic diversity. Almost half of the studies conducted in the U.S. failed to report race or do so adequately. Despite the knowledge that stroke disproportionately impacts Black adults in the U.S., White participants were disproportionately represented in most studies in our review. However, on other demographic risk factors such as age and sex, the studies on average aligned with the risk profile. Women are more likely to get stroke than men and were on average 54% of each sample. The mean age of most samples was 55–64, followed by 45–54; the two age cohorts with highest stroke prevalence.

The lack of Black participants in this research is not surprising. We know that Black participants are underrepresented in clinical trials and that medical mistrust results from decades of medical exploitation and experimentation [83]. At the same time, we risk exacerbating health disparities if we are not transformative in our intervention approaches. In the context of the digital divide, we cannot achieve health equity if we do not understand how digital health interventions may be accepted and adopted by Black Americans and other historically underrepresented groups. Community-engaged and community-driven frameworks emphasize co-creation by engaging communities early on in the development of interventions [36].

There are limitations to this review. First, we only assessed research published in the English language, therefore, other international innovative work may have been missed. Second, given publication bias, it is possible that the systematic reviews included in this larger review did not capture all research that suited their inclusion criteria, disproportionately including work with positive findings. Third, some reviews also synthesized the same research. While most of the individual studies were only cited once (78%), significant overlap may introduce bias in the strength of our findings. Fourth, aggregating outcomes in this review may mask important nuances for clinical practice (e.g., static vs. dynamic vs. walking balance). Lastly, it is possible that some interventions are not explicitly reported as multi-modal since some research included in more than one review was described differently in each review.

## Conclusion

Given the recent increase in stroke mortality, leveraging the success of digital health interventions can significantly reduce morbidity, mortality, and the financial burden of stroke, while increasing health equity. Digital interventions expand access to care while still providing a means to receive consultation, education, case management, disease monitoring, mentoring, and rehabilitation. However, interventions cannot be successful without users—whose characteristics and deployment environment must be carefully considered when designing interventions. The social and structural determinants of health, of which broadband is a newly proposed addition, are necessary to consider when planning interventions that will certainly coexist with the digital divide.

### Supplementary Information

**Additional file 1: Additional file Table 1.** Search strategy across databases.** Additional file Table 2.** Critical appraisal of included reviews.** Additional file Table 3.** Summary of included reviews.** Additional file Table 4.** Preferred Reporting Items for Systematic reviews and Meta-Analyses extension for Scoping Reviews (PRISMA-ScR) Checklist.

## Data Availability

The datasets generated and/or analysed during the current study is available from the corresponding author on reasonable request.
