# Supplementary material for: Do digital health interventions hold promise for stroke prevention and care in Black and Latinx populations in the United States? A scoping review
Source: BMC Public Health. 2023 Dec 21;23:2549. doi: 10.1186/s12889-023-17255-6 (PMC10734160; doi:10.1186/s12889-023-17255-6)
Supplement: Supplementary file 1 — Additional file 1: Additional file Table 1. Search strategy across databases. Additional file Table 2. Critical appraisal of included reviews. Additional file Table 3. Summary of included reviews. Additional file Table 4. Preferred Reporting Items for Systematic reviews and Meta-Analyses extension for Scoping Reviews (PRISMA-ScR) Checklist. [file 12889_2023_17255_MOESM1_ESM.docx]

**Appendix Table 1. Search strategy across databases**

| Database | Strategy |
| --- | --- |
| PubMed | (“stroke” [MeSH Terms] OR “hypertension” [MeSH Terms] OR “diabetes mellitus” [MeSH Terms] OR “hyperlipidemias” [MeSH Terms] OR “smoking cessation” [MeSH Terms]) |
|  | AND (“computers”[MeSH Terms] OR “computer*”[Title/Abstract] OR “Computers, Handheld”[Mesh] OR “Mobile Device*”[Title/Abstract] OR “Tablet Computers” [Title/Abstract] OR “telemedicine”[MeSH Terms] OR “telemedicine”[Title/Abstract] OR “telehealth”[Title/Abstract] OR “remote consultation”[MeSH Terms] OR (“remote” AND “consultation”) OR “teleconsultation”[Title/Abstract] OR “internet”[MeSH Terms] OR “internet”[Title/Abstract] OR “World Wide Web”[Title/Abstract] OR “cell phone”[MeSH Terms] OR “cell phone*”[Title/Abstract] OR “cellular phone*”[Title/Abstract] OR “Mobile Phone*” [Title/Abstract] OR “smartphone”[MeSH Terms] OR “smart phone*”[Title/Abstract] OR “smartphone*”[Title/Abstract] OR “mobile applications”[MeSH Terms] OR “mobile app”[Title/Abstract] OR “mobile apps”[Title/Abstract] OR "Wearable Electronic Devices"[Mesh] OR "Wearable Electronic Devices"[Title/Abstract] OR "Wearable Devices"[Title/Abstract] OR "Wearable Technology"[Title/Abstract] OR "Monitoring, Ambulatory"[Mesh] OR "Ambulatory Monitoring"[Title/Abstract] OR "Outpatient Monitoring"[Title/Abstract] OR "Wireless Technology"[Mesh] OR "Wireless Technology"[Title/Abstract] OR "Telemetry"[Mesh] OR "Telemetry"[Title/Abstract] OR "Videoconferencing"[Mesh] OR ”Videoconferenc*"[Title/Abstract] OR “Telehomecare”[Title/Abstract] OR “Telecare”[Title/Abstract] OR “Virtual”[Title/Abstract] OR “Internet-Based Intervention”[MeSH Terms] OR “Internet-Based Intervention”[Title/Abstract] OR “Internet Based Intervention”[Title/Abstract] OR “Internet-Based”[Title/Abstract] OR “Internet Based”[Title/Abstract] OR “Web based”[Title/Abstract] OR “Web-based”[Title/Abstract] OR “Mobile health”[Title/Abstract] OR (“Mobile health” AND “devices”) OR “mHealth”[Title/Abstract] OR “ehealth”[Title/Abstract] OR “Electronic Health”[Title/Abstract] OR “Social media”[Mesh] OR “Social media”[Title/Abstract] OR “Telephone”[Mesh] OR “Telephone”[Title/Abstract] OR “Computer assisted”[Title/Abstract] OR “Computer-assisted”[Title/Abstract] OR “Text messaging”[Mesh] OR “Text messaging”[Title/Abstract] OR “Text-messaging”[Title/Abstract] OR “Texting”[Title/Abstract] OR “Text Messages”[Title/Abstract] OR “Telecommunications”[Mesh] OR “Telecommunication*”[Title/Abstract] OR “Microcomputers”[Mesh] OR “Microcomputers”[Title/Abstract] OR “Minicomputers”[Mesh] OR “Minicomputers”[Title/Abstract] OR “Electronic Mail”[Mesh] OR “Electronic Mail”[Title/Abstract] OR “e-mail”[Title/Abstract] OR “email”[Title/Abstract] OR “Remote Sensing Technology”[Title/Abstract] OR “Telerehabilitation”[Mesh] OR “Telerehab*”[Title/Abstract] OR “Remote Rehabilitation”[Title/Abstract] OR (“Remote” AND “Rehabilitation”) OR “Tele-rehabilitation”[Title/Abstract] OR “Virtual Rehabilitation”[Title/Abstract] OR (“Virtual” AND “Rehabilitation”) OR “Telecoaching”[Title/Abstract] OR “Video consultation”[Title/Abstract] OR (“Video” AND “consultation”) OR “Patient Portals”[Mesh] OR “Patient Internet Portal*”[Title/Abstract] OR “Patient Portal*”[Title/Abstract] OR “Patient Web Portal*”[Title/Abstract] OR “Video Games”[Mesh] OR “Video Games”[Title/Abstract] OR “Computer Games”[Title/Abstract] OR “Electronic Health Records”[Mesh]) |
|  | AND ("Clinical Trial"[Publication Type] OR "Clinical Trials as Topic"[Mesh] OR "Evaluation Study"[Publication Type] OR "Randomized Controlled Trial"[Publication Type] OR "Randomized Controlled Trials as Topic"[Mesh] OR “Comparative Study”[Publication Type] OR "Prospective Studies"[Mesh] OR "Longitudinal Studies"[Mesh] OR "Treatment Outcome"[Mesh] OR "Follow-Up Studies"[Mesh] OR “quasi-experimental”[Title/Abstract] OR “quasi experimental”[Title/Abstract] OR (“quasi” AND “experimental”)) |
|  | AND ("Systematic Reviews as Topic"[Mesh] OR "Systematic Review"[Publication Type] OR "Meta-Analysis as Topic"[Mesh] OR "Meta-Analysis"[Publication Type]) |
| Web Of Science^a^ | (stroke OR hypertension OR diabetes mellitus OR hyperlipidemias OR smoking cessation) |
|  | AND (computer* OR telemedicine OR telehealth OR remote consultation OR internet OR cell phone* OR smartphone* OR mobile application* OR ehealth OR mhealth OR mobile device* OR mobile phone* OR tablet computer* OR teleconsultation OR world wide web OR wearable device* OR wearable electronic device* OR wearable technology OR ambulatory monitoring OR telemetry OR videoconferenc* OR video conferenc* OR telecare OR telehomecare OR virtual OR web based OR web-based OR social media OR telephone* OR texting OR text messag* OR computer assisted OR electronic health OR telecommunication* OR email OR e-mail OR electronic mail OR remote sensing technology OR telerehab* OR remote rehab* OR video consultation OR telecoaching OR patient portal* OR video game*) |
|  | AND (randomized controlled trial OR randomized clinical trial OR clinical trial OR follow up OR longitudinal OR prospective OR experimental OR quasi experiment OR intervention OR evaluation) |
|  | AND (systematic review OR meta analysis) |
| EMBASE | ('stroke':ab,ti OR 'hypertension':ab,ti OR 'diabetes':ab,ti OR 'hyperlipidemias':ab,ti OR 'smoking cessation program':ab,ti) |
|  | AND ('computer'/exp OR computer OR 'mobile phone'/exp OR 'mobile phone' OR 'smartphone'/exp OR smartphone OR 'tablet computer'/exp OR 'tablet computer' OR 'telemedicine'/exp OR telemedicine OR 'telehealth'/exp OR telehealth OR 'teleconsultation'/exp OR teleconsultation OR 'web-based intervention'/exp OR 'web-based intervention' OR 'mobile application'/exp OR 'mobile application' OR 'wearable computer'/exp OR 'wearable computer' OR 'ambulatory monitoring'/exp OR 'ambulatory monitoring' OR 'telerehabilitation'/exp OR telerehabilitation OR 'virtual reality'/exp OR 'virtual reality' OR 'videoconferencing'/exp OR videoconferencing OR 'video game'/exp OR 'video game' OR 'electronic medical record'/exp OR 'electronic medical record' OR 'electronic device'/exp OR 'electronic device' OR 'medical record'/exp OR 'medical record' OR 'telecommunication'/exp OR telecommunication OR 'telemetry'/exp OR telemetry OR 'telemonitoring'/exp OR telemonitoring OR 'social media'/exp OR 'social media' OR 'text messaging'/exp OR 'text messaging' OR 'e mail'/exp OR 'e mail' OR 'remote sensing'/exp OR 'remote sensing') |
|  | AND ('randomized controlled trial':ab,ti OR 'clinical study':ab,ti OR 'quasi experimental study':ab,ti OR 'intervention study':ab,ti OR 'evaluation study':ab,ti OR 'longitudinal study':ab,ti OR 'follow up':ab,ti OR 'prospective study':ab,ti) |
|  | AND ('systematic review':ab,ti OR 'meta analysis':ab,ti) |

Note. Additional filtering of the English language was done. Publication dates from January 1, 2015-June 8, 2021 were included. Reproducibility may vary if additional articles were indexed after the search.

^a^The same strategy was conducted twice, joined by an ‘OR’ statement. Each strategy was preceded by either “TI=” or “AB=”

**Appendix Table 2. Critical appraisal of included reviews**

| **First Author, Year** | **Is the review question clearly and explicitly stated?** | **Were the inclusion criteria appropriate for the review question?** | **Was the search strategy appropriate?** | **Were the sources and resources used to search for studies adequate?** | **Were the criteria for appraising studies appropriate?** | **Was critical appraisal conducted by two or more reviewers independently?** | **Were the methods used to combine studies appropriate?** | **Was the likelihood of publication bias assessed?** | **Were recommendations for policy and/or practice supported by the reported data?** | **Were the specific directives for new research appropriate?** |
| --- | --- | --- | --- | --- | --- | --- | --- | --- | --- | --- |
| Afzal, 2015 | + | + | + | + | + | + | + | - | + | + |
| Baratloo, 2018 | + | + | + | + | + | + | + | + | + | + |
| Beishuizen, 2016 | + | + | + | + | + | + | + | + | + | + |
| Bingham, 2021 | + | + | + | + | + | + | + | + | + | + |
| Blok, 2020 | + | + | + | + | + | + | + | + | + | + |
| Celik, 2020 | + | + | + | + | + | + | + | + | + | + |
| Chen L, 2016 | + | + | + | + | + | + | + | - | + | + |
| Cheok, 2015 | + | + | + | + | + | + | + | + | + | + |
| Choi, 2020 | + | + | + | + | + | + | + | - | + | + |
| Corbetta, 2015 | + | + | + | + | + | + | + | + | + | + |
| Dening, 2020 | + | + | + | + | + | + | + | + | + | + |
| Enricho Nkhoma, 2021 | + | + | + | + | + | - | + | + | + | + |
| Farmer, 2016 | + | + | + | + | + | + | + | + | + | + |
| Freund, 2021 | + | + | + | + | + | + | + | + | + | + |
| Fu, 2017 | + | + | + | + | + | + | + | - | + | + |
| Gandapur, 2016 | + | + | + | + | - | + | + | - | + | + |
| Gibbons, 2016 | + | + | + | + | - | + | + | + | + | + |
| Holmen, 2017 | + | + | + | + | + | + | + | + | + | + |
| Howland, 2021 | + | + | + | + | + | + | + | - | + | + |
| Huang, 2019 | + | + | + | + | + | + | + | + | + | + |
| Hyun, 2021 | + | + | + | + | + | + | + | - | + | + |
| Islam, 2020 | + | + | + | + | + | + | + | + | + | + |
| Li Z, 2016 | + | + | + | + | + | - | + | - | + | + |
| Lieber, 2015 | + | + | - | + | + | - | + | + | + | + |
| Lunde, 2018 | + | + | + | + | + | + | + | - | + | + |
| McCrabb, 2019 | + | + | + | + | + | + | + | + | + | + |
| McLean, 2016 | + | - | + | + | + | + | + | + | + | + |
| Mekbib, 2020 | + | + | + | + | + | - | + | + | + | + |
| Mohammadi, 2019 | + | + | + | + | + | + | + | - | + | + |
| Mushcab, 2015 | + | + | + | + | + | - | + | - | + | + |
| Niemeijer, 2020 | + | + | + | + | + | + | + | - | + | + |
| Palma, 2017 | + | + | + | + | + | + | + | + | + | + |
| Parker, 2020 | + | + | + | + | + | + | + | - | + | + |
| Seo, 2015 | + | + | + | + | + | - | + | + | + | + |
| Taylor, 2017 | + | + | + | + | + | + | + | + | + | + |
| Waseem, 2021 | + | - | + | + | + | - | + | + | - | + |
| Xie, 2020 | + | + | + | + | - | + | - | + | + | + |
| Zhuang, 2020 | - | + | - | + | + | + | + | + | + | + |

**Appendix Table 3. Summary of included reviews**

| **First Author, Year** | **Target Population** | **Modality** | **Tele-function** | **Search Range** | **Design of Included Studies** | **Number of studies conducted in the United States (%)** | **Outcomes** | **Results** |
| --- | --- | --- | --- | --- | --- | --- | --- | --- |
| Afzal, 2015 | Survivors of cryptogenic stroke or transient ischemic attack, ≥ 18 years old | Health devices | Monitoring | As of 2014 | 3 RCT, 13 observational | 5/16 (31.3%) | Atrial Fibrillation detection | **Meta-analysis:** Pooled odds ratio (OR) of identifying AF in RCTs showed increased detection of AF with prolonged monitoring (OR 4.54, 95% confidence interval [CI] 2.92, 7.06; P < 0.00001) compared to routine outpatient follow-up. Overall detection of AF with outpatient monitoring was 17.6% (CI: 12.5–22.7). There was significantly higher AF detection with ILR (23.3%; CI: 13.83–32.29) compared to wearable devices (13.6%; CI: 7.91–19.32; P < 0.05). |
| Baratloo, 2018 | Survivors of acute ischemic stroke | Telestroke | Consultation | As of May 2017 | 26 observational | 12/26 (46.2%) | Mortality, Symptomatic Intracranial hemorrhage, Onset to door/on set to treatment, length of hospital stay | **Meta-analysis:** No significant differences between the telestroke and control groups in terms of in-hospital mortality (OR = 1.21, 95% CI [0.98, 1.49]), 90-day mortality (OR = 1.08, 95% CI [0.85, 1.37]), symptomatic intracranial hemorrhage (sICH) (OR = 1.10, 95% CI [0.79, 1.53]), and favorable clinical outcome at discharge (OR = 1.03, 95% CI [0.69, 1.53]) and 90 days later (OR = 0.99, 95% CI [0.82, 1.18]). The onset-to-door (OTD) duration (MD = −10.4 minutes, 95% CI [−14.79, −0.01]) and length of hospital stay (MD = −0.55 days, 95% CI [−1.02, −0.07]) were significantly shorter in the telestroke group, compared to the control group. |
| Beishuizen, 2016 | Adults who have a mixed level of cardiovascular risk (one or more cardiovascular risk factors or established cardiovascular disease), ≥ 50 years old | Web-based | Case Management | 1995 - 2015 | 57 RCT | 39/57 (68.4%) | BP, HbA1c, LDL cholesterol, smoking status, weight, level of physical exercise, or a composite cardiovascular risk score | **Meta-analysis:** A significant reduction in systolic blood pressure (mean difference –2.66 mmHg, 95% CI –3.81 to –1.52), diastolic blood pressure (mean difference –1.26 mmHg, 95% CI –1.92 to –0.60), HbA1c level (mean difference –0.13%, 95% CI –0.22 to –0.05), LDL cholesterol level (mean difference –2.18 mg/dL, 95% CI –3.96 to –0.41), weight (mean difference –1.34 kg, 95% CI –1.91 to –0.77), and an increase of physical activity (standardized mean difference 0.25, 95% CI 0.10-0.39) in the Web-based intervention group was found. |
| Bingham, 2021 | Adult patients prescribed medications for chronic condition management (e.g., type 2 diabetes, hypertension, and/or dyslipidemia) | Phone-Based, Messaging, Mobile Application (not in combination) | Education, Monitoring, Case Management | January 1, 1998 - December 31, 2019 | 5 pre-post intervention, 4 RCT, 4 observational | 10/13 (76.9%) | Medication adherence | The systematic review demonstrated that electronic health (eHealth) and telehealth interventions were successful at improving medication adherence, whereas mobile health interventions did not improve medication adherence. |
| Blok, 2020 | Adults with hypertension | Health devices | Monitoring | As of April 2020 | 15 RCT | 8/15 (53.3%) | Systolic Blood Pressure | **Meta-analysis:** Overall, the incremental decrease in systolic blood pressure using eHealth, compared to usual care, was 3.87 95% confidence interval (CI) 2.98–4.77) mmHg at 6 months and 5.68 (95% CI 4.77–6.59) mmHg at 12 months follow-up. |
| Celik, 2020 | Adults 50-65 years old, with type 2 diabetes | Web-Based | Education, Monitoring, Case Management | As of April 2019 | 8 RCT | 5/8 (62.5%) | HbA1c, blood pressure, and total cholesterol | **Meta-analysis:** Online interventions resulted in the improvement of HbA1c (pooled mean difference on HbA1c: -0.35%, 95% CI (-0.52, -0.18), P<0.001). |
| Chen L, 2016 | Adults with hemiparesis following stroke during acute, subacute, and chronic phases, ≥ 18 years old | Virtual Reality | Rehabilitation | January 2006- December 2015 | 9 RCT | 1/9 (11/1%) | Static balance, dynamic balance, walking balance, gait, and mobility | All studies, except one, showed significant improvement in static or dynamic balance outcomes group. Moderate evidence that virtual reality training is an effective adjunct to standard rehabilitation program. |
| Cheok, 2015 | Stroke survivors with stroke of any chronicity | Virtual Reality | Rehabilitation | As of July 2014 | 6 RCT | 0/6 (0%) | Functional mobility, balance | **Meta-analysis:** The addition of Wii to conventional rehabilitation resulted in significant mean differences in favor of additional Wii compared with standard care for Timed Up and Go test (TUG) (0.81 points, CI 0.29-1.33, P = .002), but not for other mobility and functional outcomes: Functional Independence Measure (FIM) score (0.45, CI 0.21-1.11, P = .18), Berg Balance Score (0.64, CI -3.66 to 2.39, P = .68), anteroposterior postural sway (0.23, CI 0.38-0.84, P = .46). The addition of Wii gaming to conventional rehabilitation in patients with chronic stroke significantly improved performance in TUG and not in the other physical measures. The pooled effect was small and not beyond the minimal detectable change. |
| Choi, 2020 | Adults with hypertension and ischemic heart disease ≥ 18 years old | Mobile Application | Education, Monitoring, Case Management | January 2006 - August 2017 | 3 NCRT, 9 RCT, 4 pre-post, 1 ITS | 6/17 (35.3%) | Blood Pressure, BMI, Medication adherence | Two of three studies showed a significant decrease of body mass index in patients with ischemic heart disease after the app-based interventions compared to the control group. Five of seven studies reported a significant change in medication adherence. |
| Corbetta, 2015 | Adults with ischemic or hemorrhagic stroke, without other pathological conditions affecting lower limbs, > 18 years old | Virtual Reality | Rehabilitation | As of 2014 | 15 RCTs | 2/15 (13.3%) | Walking speed, balance, mobility | **Meta-analysis:** When VRBR replaced some or all of the standard rehabilitation, there were statistically significant benefits in walking speed (MD 0.15 m/s, 95% CI 0.10 to 0.19), balance (MD 2.1 points on the Berg Balance Scale, 95% CI 1.8 to 2.5) and mobility (MD 2.3 seconds on the Timed Up and Go test, 95% CI 1.2 to 3.4). When VRBR was added to standard rehabilitation, mobility showed a significant benefit (0.7 seconds on the Timed Up and Go test, 95% CI 0.4 to 1.1), but insufficient evidence was found to comment about walking speed (one trial) and balance (high heterogeneity). Substituting some or all of a standard rehabilitation regimen with VRBR elicits greater benefits in walking speed, balance and mobility in people with stroke. |
| Dening, 2020 | Adults ≥ 18 years old, with type 2 diabetes | Web-Based | Case Management | January 2013 - May 2019 | 5 RCT | 3/5 (60%) | Dietary behavior change, HbA1c, fasting blood sugar, waist circumference | Significant improvements in dietary behavior were reported in four out of the five studies, representing healthier food choices, improvements in eating habits, reductions in carbohydrates, added sugar, sodium, saturated fat and overall fat intake, and/or increases in dietary knowledge. Three studies found significant mean reductions for hemoglobin A1c ranging from –0.3% to –0.8%, and/or weight ranging from –2.3 kg to –12.7 kg, fasting blood glucose (–1 mmol/L), waist circumference (–1 cm), and triglycerides (–60.1 mg/dL). |
| Enricho Nkhoma, 2021 | Adults ≥ 18 years old, with type 2 diabetes | Mobile Application | Education, Case Management | 2013 - 2020 | 6 RCTs | 0/6 (0%) | Medication adherence, HbA1c, BMI | **Meta-analysis:** At 3 months, DSMES apps proved effective in improving medication adherence (standardized mean difference (SMD)=0.393, 95% CI 0.17 to 0.61), glycated hemoglobin (HbA1c) (mean difference (MD)=−0.314, 95% CI −0.477 to –0.151) and Body Mass Index (BMI) (MD=−0.28, 95% CI −0.545 to –0.015). All pooled estimates had low heterogeneity (I2=0%). DSMES apps had significant small to moderate effects on medication adherence, HbA1c, and BMI of patients with T2D compared with usual care. |
| Farmer, 2016 | Adults ≥ 18 years old, with type 2 diabetes | Messaging, Health device & Messaging in Combination, Web-based & Messaging in Combination | Monitoring, Case Management | 1990 - 2014 | 10 RCT, 1 cross-over | 6/11 (54.5%) | Medication adherence | A **meta-analysis** of five trials (eight interventions) combining monitoring and messaging strategies showed that the pooled difference in medication adherence between intervention and control was moderate and not statistically significant [standardized mean difference = 0.22 (95% CI 0.05; 0.49)]. |
| Freund, 2021 | Stroke survivors ≥ 18 years old, who had experienced a stroke or transient ischemic attack and were residing in the community or support persons ≥ 18 and identified as the primary support for a person who had experienced a stroke or transient ischemic attack | Computer-delivered, Web-based, Mobile Applications, Messaging (not in combination) | Education, Monitoring, Case Management, Rehabilitation | 2006 - June 19, 2020 | 15 RCT, 2 NRCT | 2/17 (11.8%) | Stroke outcomes (e.g., speech, hemiparesis, cognition), BMI, blood pressure, HbA1c, smoking, diet, medication adherence, physical activity | Fifteen studies reported significant positive effects for at least one outcome examined including stroke-specific outcomes, physical outcomes, and behavioral outcomes. ICT-based interventions are likely to provide benefit to stroke survivors and their support persons, *regardless* of the modality of delivery. |
| Fu, 2017 | Adults ≥ 18 years old, with type 2 diabetes | Mobile Applications in *Combination* with Web-based, health devices, and messaging | Education, Monitoring, Case Management | January 1, 2011- January 17, 2017 | 6 RCT, 4 pre-post, 7 observational | 6/17 (35.3%) | HbA1c | Clinical effectiveness, measured by reductions in HbA1c, ranged from 0.15% to 1.9%. |
| Gandapur, 2016 | Adults with hypertension, ischaemic heart disease, myocardial infarction, acute coronary syndrome, heart failure, stroke, or peripheral arterial disease | Messaging, Health device + Smartphone (Call or text) in Combination, Phone-based, Mobile application | Education, Case Management | January 1, 1966 - December 11, 2015 | 10 RCT | 5/10 (50%) | Medication adherence | All 10 studies reported that mHealth interventions improved medication adherence, though the magnitude of benefit was not consistently large and in one study was not greater than a telehealth comparator. |
| Gibbons, 2016 | Stroke survivors ≥ 18 years old, with functional loss varying from acute to chronic | Virtual Reality | Rehabilitation | As of August 2015 | 22 RCTs | 1/22 (4.5%) | Functional balance, static balance, functional mobility, spatiotemporal characteristics/kinematics of gait, motor function, muscle tone | **Meta-analysis:** Significant differences in favor of VR group were found for functional balance (SMD 0.42, 95% CI 0.11–0.73), gait velocity (WMD 0.12, 95% CI 0.03–0.22), cadence (WMD 11.91, 95% CI 2.05–21.78), and stride length (WMD 9.79, 95% CI 0.74–18.84) within the chronic population. VR improves functional balance and various aspects of gait in chronic populations. |
| Holmen, 2017 | Persons with diabetes ≥ 16 years old | Mobile Application | Education, Monitoring, Case Management | 2008 - 2016 | 4 RCTs, 1 matched controlled design, 1 experimental design that randomly selected participants and assigned them in two groups | 0/6 (0%) | HbA1c, SBP, DBP | A statistically significant improvement in the primary measure of outcome was found in 3 of the 6 included studies. |
| Howland, 2021 | Adults ≥ 18 years old, with type 2 diabetes | Web-based, Mobile application, Health device + Web-based in Combination, Web-based + Mobile app (optional) in Combination, Web & Mobile *phone* in Combination (unclear if phone-based or Messaging), Mobile App & Messaging in Combination | Education, Monitoring, Case Management, Mentoring | As of October 2019 | 13 RCT, 2 quasi-experimental, 2 mixed methods (1 with RCT, 1 with quasi) | 5/17 (29.4%) | Physical activity, HbA1c | Significant improvements in physical activity and sedentary behavior were identified in web and mobile phone-based interventions. Modest improvements in glycemic control were reported. |
| Huang, 2019 | Adults ≥ 19 years old | Phone-based, Web-based (not in combination) | Education, Monitoring, Case Management | As of August 31, 2014 | 25 RCT | 12/25 (48%) | BMI | **Meta-analysis:** Significant differences in body mass index changes (pooled difference in means = -0.49, 95% confidence interval -0.63 to -0.34, p < 0.001) between the telemedicine and control groups. The subgroup analyses found that either Internet-based or telephone-based intervention was associated with greater changes in body mass index than in controls. |
| Hyun, 2021 | Adults ≥ 18 years old, with type 2 diabetes | Mobile Application, Combination: Mobile Apps + "ecoaching" (Ecoaching via messaging, mobile app, or phone calls) | Monitoring, Case Management | As of October 2020 | 14 RCTs | 1/14 (7.1%) | HbA1c, fasting blood sugar | **Meta-analysis:** Mobile apps or apps with e-coaching interventions for T2DM patients were more effective in improving the HbA1c values and FBS, than usual care. |
| Islam, 2020 | Adults ≥ 18 years old, and children | Mobile Application | Education, Monitoring, Case Management | January 1, 2000 - April 30, 2019 | 11 RCT, 1 case-control | 4/12 (33.3%) | Weight loss, BMI, Physical activity | **Meta-analysis:** Compared with the control group, the use of a mobile phone app was associated with significant changes in body weight (−1.07 kg, 95% CI −1.92 to −0.21, P=.01) and body mass index (−0.45 kg/m2, 95% CI −0.78 to −0.12, P=.008). Moreover, a nonsignificant increase in physical activity was observed (0.17, 95% CI −2.21 to 2.55, P=.88). |
| Li Z, 2016 | Stroke survivors | Virtual Reality | Rehabilitation | As of May 2015 | 16 RCT | 0/16 (0%) | Balance, Force Platform Indicators (such as center of pressure measures, sway velocity, stability index, weight distribution), adverse events and therapy adherence. | **Meta-analysis:** People who received virtual reality interventions showed marked improvements in Berg Balance Scale (mean difference: 1.46, 95% confidence interval: 0.09–2.83, P<0.05, I2=0%) and Timed Up and Go Test (mean difference: –1.62, 95% confidence interval: –3.07– –0.16, P<0.05, I2=24%) compared with controls. |
| Lieber, 2015 | Persons with type 1 or type 2 diabetes | Health devices | Monitoring, Case Management | As of November 2013 | 5 RCT | 3/5 (60%) | HbA1c | **Meta-analysis:** Four of the five studies showed a greater reduction in HbA1c in the intervention group compared to controls at 6 months, although only one was statistically significant. There was considerable heterogeneity between studies (I2 = 69.5%, p = 0.02), and the random effects model estimated the aggregate effect size for mean difference in reduction of HbA1c levels to be 0.08% (95% confidence interval 0.12% to 0.28%), which was not statistically significant (p = 0.42). |
| Lunde, 2018 | Adults diagnosed with cardiovascular diseases, cancers, chronic pulmonary diseases, or diabetes mellitus, ≥ 18 years old | Mobile Application | Monitoring, Case Management | As of February 23, 2017 | 9 RCT | 2/9 (22.2%) | HbA1c, body weight, waist circumference | **Meta-analysis:** Statistical significant effect was shown in HbA1c in 5 of 8 studies, as well in body weight in one of 5 studies and in waist circumference in one of 3 studies evaluating these outcomes. Seven of the included studies were included in the **meta-analyses** and demonstrated significant overall effect on HbA1c on a short term (3-6 months; P=.02) with low heterogeneity (I=41%). In the long term (10-12 months), the overall effect on HbA1c was statistically significant (P=.009) and without heterogeneity (I=0%). |
| McCrabb, 2019 | Current smokers ≥ 18 years old | Web-Based | Case Management, Mentoring | As of September 2017 | 45 RCT | 27/45 (60%) | Smoking cessation | **Meta-analysis:** Intervention effectiveness was found in the short term for all outcome measures (OR = 1.29, 95% CI 1.12, 1.50, p = .001), for “prolonged abstinence” (OR = 1.43, 95% CI 1.09, 1.87, p = .009), and “30 day PPA” (OR = 1.75, 95% CI 1.13, 2.72, p = .013). Internet-based programs were effective in the long term for all outcome measures (OR = 1.19, 95% CI = 1.06, 1.35, p = .004) and for “prolonged abstinence” (OR = 1.40, 95% CI 1.19, 1.63, p < .001). On average, interventions used more BCTs than comparison groups (6.6 vs. 3.1, p = .0002). Internet-based smoking cessation interventions increased the odds of cessation by 29 percent in the short term and by 19 percent in the long term. |
| McLean, 2016 | Adults with hypertension ≥ 18 years old | Web-based, Phone-based, Mobile application | Education, Monitoring, Case Management | Not Stated | 7 RCT | 3/7 (42.9%) | Blood Pressure (SBP, DBP), Self-efficacy, medication adherence, dietary change, physical activity | **Meta-analysis:** (Interactive digital interventions) IDIs lower both SBP and DBP compared to usual care. IDIs significantly reduced SBP, with the weighted mean difference being 3.74 mmHg [95% confidence interval (CI) 2.19 to 2.58] with no heterogeneity observed. For DBP, four out of six studies indicated a greater reduction for intervention compared to controls, with no difference found for two. For DBP, a significant reduction of 2.37 mmHg (95% CI 0.40 to 4.35) was found, but considerable heterogeneity was noted (I-squared 1⁄4 80.1%, P 1⁄4 <0.001). |
| Mekbib, 2020 | First-time stroke survivors without other comorbidities | Virtual Reality | Rehabilitation | 2010 - February 2019 | 27 RCT | 3/27 (11/1%) | Body Functions (joints, bones, muscles, pain, mental functioning, additional sensory functions, Activity (changing and maintaining body position, self-care, communicating, carrying, moving, handling objects) and participation (household tasks, interpersonal interactions, civic life, work and employment) | **Meta-analysis:** The analysis indicated that the VR group showed statistically significant improvement in the recovery of UL function versus the control group. |
| Mohammadi, 2019 | Stroke survivors aiming to improve balance control, all stages of stroke (acute, subacute, chronic) | Virtual Reality | Rehabilitation | January 2000 - August 2017 | 14 RCT | 0/14 (0%) | Balance | Among the high quality studies, significant between-group improvement favoring virtual reality in combination with conventional therapy was found compared to conventional therapy alone. Thirteen homogenous (n = 348, I2 = 37.6%, P = .083) studies were included in the **meta-analysis**. Significant improvement was observed in the experimental group compared to control group with a medium effect size of .64, confidence interval of .36-.92. |
| Mushcab, 2015 | Adults ≥ 18 years old, with type 2 diabetes prescibed insulin treatment | Health devices | Monitoring, Case Management | Not Stated | 9 RCT, 10 quasi-experimental | 5/19 (26.3%) | HbA1c | Fifteen studies showed positive improvement in HbA1c levels. |
| Niemeijer, 2020 | Stroke survivors with symptoms of cognitive impairment | Computer-delivered | Rehabilitation | As of March 17, 2019 | 5 RCT | 0/5 (0%) | Working memory after stroke | **Meta-analysis:** There is insufficient evidence to conclude if CBCR is beneficial for patients with working memory deficits after stroke. |
| Palma, 2017 | Stroke survivors of all stages | Virtual Reality | Rehabilitation | As of June 16, 2015 | 54 RCTs (but only 20 in this article, 34 of lower quality in supplementary material) | 1/20 (5%) | Body Functions (joints, bones, muscles, pain, mental functioning, additional sensory functions Activity (changing and maintaining body position, self-care, communicating, carrying, moving, handling objects) and participation (household tasks, interpersonal interactions, civic life, work and employment) | The effects of virtual reality on stroke rehabilitation based on the ICF framework are positive in Body Function and Body Structure. However, the results in the domains Activity and Participation are inconclusive. |
| Parker, 2020 | Stroke survivor ≥ 18 years old | Health devices | Rehabilitation | 2000 - April 2019 | 7 RCTs, 4 comparison trials | 5/11 (45.5%) | Functional gain, participation | Only 1 study found significant between-group differences for systems functioning and activity (P≤.02). |
| Seo, 2015 | Adults ≥ 18 years old | Web-Based | Monitoring, Case Management, Mentoring | 1980 - April 2014 | 31 RCT | 8/31 (25.8%) | Mean waist circumference | Internet-based interventions showed a significant reduction in waist circumference (mean change –2.99 cm, 95% CI −3.68 to −2.30, I=93.3%) and significantly better effects on waist circumference loss (mean loss 2.38 cm, 95% CI 1.61-3.25, I=97.2%) than minimal interventions such as information-only groups. **Meta-regression** results showed that baseline waist circumference, gender, and the presence of social support in the intervention were significantly associated with waist circumference reduction. |
| Taylor, 2017 | Current smokers, any age | Web-Based | Education, Case Management, Mentoring | As of August 23, 2016 | 65 RCT | 35/65 (53.8%) | Smoking cessation of at least 6 months duration | **Meta-analysis:** Pooled results demonstrated an effect in favor of the intervention (RR 1.15, 95% CI 1.01 to 1.30, n = 6786). However, statistical heterogeneity was high (I2=58%). Five trials compared an Internet intervention to an active control. The pooled effect estimate favored the control group, but crossed the null (RR 0.92, 95% CI 0.78 to 1.09, n = 3806, I2=0%). Five studies evaluated an Internet program plus behavioral support compared to a non-active control (n = 2334). Pooled, these studies indicated a positive effect of the intervention (RR 1.69, 95% CI 1.30 to 2.18). Although statistical heterogeneity was substantial (I2 = 60%). Four studies evaluated the Internet plus behavioral support compared to active control. None of the studies detected a difference between trial arms (RR 1.00, 95% CI 0.84 to 1.18, n = 2769, I2 = 0%). Seven studies compared an interactive or tailored Internet intervention, or both, to an Internet intervention that was not tailored/interactive. Pooled results favored the interactive or tailored program, but the estimate crossed the null (RR 1.10, 95% CI 0.99 to 1.22, n = 14,623, I2 = 0%). Three studies compared tailored with non-tailored Internet-based messages, compared to non-tailored messages. The tailored messages produced higher cessation rates compared to control, but the estimate was not precise (RR 1.17, 95% CI 0.97 to 1.41, n = 4040), and there was evidence of unexplained substantial statistical heterogeneity (I2 = 57%). The evidence from trials in adults suggests that interactive and tailored Internet-based interventions with or without additional behavioral support are moderately more effective than non-active controls at six months or longer, but there was no evidence that these interventions were better than other active smoking treatments. However, some of the studies were at high risk of bias, and there was evidence of substantial statistical heterogeneity. |
| Waseem, 2021 | Adults diagnosed with AIS and treated with IV-tPA at a spoke hospital without subsequent transfer to a hub hospital (drip-and-stay) | Telestroke | Consultation | As of October 2019 | 10 non-randomized observational cohort studies with control group | 7/10 (70%) | Functionality (mRS score), 90 day Mortality | **Meta-analysis:** No significant difference in functional outcomes (mRS 0-1) (6 studies, RR = 1.09, 95% CI 0.98-1.22, p = 0.123), sICH (8 studies, RR = 0.98, 95% CI 0.64-1.51, p = 0.942), or 90-day mortality (5 studies, RR = 0.98, 95% CI 0.73-1.32, p = 0.911, respectively) between patients treated in a drip-and-stay model compared to patients treated in drip-and-ship or hub models. There was no significant heterogeneity in these outcomes. |
| Xie, 2020 | Pregnant women with gestational diabetes | Mobile application, Messaging, Web-Based (not in combination) | Education, Monitoring, Case Management, Mentoring | As of July 31, 2019 | 32 RCT | 2/32 (6.3%) | HbA1C, Fasting blood glucose, PIH or Preeclampsia | **Meta-analysis:** Significant mean reduction in of HBA1C and FBG were noted. The meta-analysis showed that telemedicine group had significant improvements in controlling glycated hemoglobin (HbA1c) [mean difference (MD) = −0.70, P < 0.01], fasting blood glucose (FBG) (MD = -0.52, P < 0.01). In the telemedicine group, lower incidences of pregnancy-induced hypertension or preeclampsia (RR = 0.48, P < 0.01). |
| Zhuang, 2020 | Adults with type 2 diabetes | Messaging | Education, Case Management | As of March 26, 2019 | 6 RCT, 4 pre-post | 2/10 (20%) | HbA1c, Medication adherence | **Meta-analysis:** SMS intervention was effective for HbA1c level and medication adherence for T2DM over first 6 months. |

Note: Results in this table pertain to the analysis conducted in each respective review and may differ from this paper's qualitative assessment and synthesis.

**Appendix Table 4. Preferred Reporting Items for Systematic reviews and Meta-Analyses extension for Scoping Reviews (PRISMA-ScR) Checklist**

| **SECTION** | **ITEM** | **PRISMA-ScR CHECKLIST ITEM** | **REPORTED ON PAGE #** |
| --- | --- | --- | --- |
| **TITLE** | | | |
| Title | 1 | Identify the report as a scoping review. | 1 |
| **ABSTRACT** | | | |
| Structured summary | 2 | Provide a structured summary that includes (as applicable): background, objectives, eligibility criteria, sources of evidence, charting methods, results, and conclusions that relate to the review questions and objectives. | 2 |
| **INTRODUCTION** | | | |
| Rationale | 3 | Describe the rationale for the review in the context of what is already known. Explain why the review questions/objectives lend themselves to a scoping review approach. | 3-4 |
| Objectives | 4 | Provide an explicit statement of the questions and objectives being addressed with reference to their key elements (e.g., population or participants, concepts, and context) or other relevant key elements used to conceptualize the review questions and/or objectives. | 4-5 |
| **METHODS** | | | |
| Protocol and registration | 5 | Indicate whether a review protocol exists; state if and where it can be accessed (e.g., a Web address); and if available, provide registration information, including the registration number. | n/a |
| Eligibility criteria | 6 | Specify characteristics of the sources of evidence used as eligibility criteria (e.g., years considered, language, and publication status), and provide a rationale. | 5-6 |
| Information sources | 7 | Describe all information sources in the search (e.g., databases with dates of coverage and contact with authors to identify additional sources), as well as the date the most recent search was executed. | 5 |
| Search | 8 | Present the full electronic search strategy for at least 1 database, including any limits used, such that it could be repeated. | 40-41 |
| Selection of sources of evidence | 9 | State the process for selecting sources of evidence (i.e., screening and eligibility) included in the scoping review. | 6-7 |
| Data charting process | 10 | Describe the methods of charting data from the included sources of evidence (e.g., calibrated forms or forms that have been tested by the team before their use, and whether data charting was done independently or in duplicate) and any processes for obtaining and confirming data from investigators. | 7-8 |
| Data items | 11 | List and define all variables for which data were sought and any assumptions and simplifications made. | 7-8 |
| Critical appraisal of individual sources of evidence | 12 | If done, provide a rationale for conducting a critical appraisal of included sources of evidence; describe the methods used and how this information was used in any data synthesis (if appropriate). | 9 |
| Synthesis of results | 13 | Describe the methods of handling and summarizing the data that were charted. | 8-9 |
| **RESULTS** | | | |
| Selection of sources of evidence | 14 | Give numbers of sources of evidence screened, assessed for eligibility, and included in the review, with reasons for exclusions at each stage, ideally using a flow diagram. | 9 |
| Characteristics of sources of evidence | 15 | For each source of evidence, present characteristics for which data were charted and provide the citations. | 10-12, 16-17 |
| Critical appraisal within sources of evidence | 16 | If done, present data on critical appraisal of included sources of evidence (see item 12). | 9-10 |
| Results of individual sources of evidence | 17 | For each included source of evidence, present the relevant data that were charted that relate to the review questions and objectives. | 10-12 |
| Synthesis of results | 18 | Summarize and/or present the charting results as they relate to the review questions and objectives. | 12-17 |
| **DISCUSSION** | | | |
| Summary of evidence | 19 | Summarize the main results (including an overview of concepts, themes, and types of evidence available), link to the review questions and objectives, and consider the relevance to key groups. | 17-21 |
| Limitations | 20 | Discuss the limitations of the scoping review process. | 21 |
| Conclusions | 21 | Provide a general interpretation of the results with respect to the review questions and objectives, as well as potential implications and/or next steps. | 22 |
| **FUNDING** | | | |
| Funding | 22 | Describe sources of funding for the included sources of evidence, as well as sources of funding for the scoping review. Describe the role of the funders of the scoping review. | 23 |

*From:* Tricco AC, Lillie E, Zarin W, O'Brien KK, Colquhoun H, Levac D, et al. PRISMA Extension for Scoping Reviews (PRISMAScR): Checklist and Explanation. Ann Intern Med. 2018;169:467–473. [doi: 10.7326/M18-0850](http://annals.org/aim/fullarticle/2700389/prisma-extension-scoping-reviews-prisma-scr-checklist-explanation).
